# Ethidium bromide transport across *Mycobacterium smegmatis *cell-wall: correlation with antibiotic resistance

**DOI:** 10.1186/1471-2180-11-35

**Published:** 2011-02-18

**Authors:** Liliana Rodrigues, Jorge Ramos, Isabel Couto, Leonard Amaral, Miguel Viveiros

**Affiliations:** 1 Unit of Mycobacteriology, Instituto de Higiene e Medicina Tropical, Universidade Nova de Lisboa, Rua da Junqueira 100, 1349-008 Lisboa, Portugal; 2 UPMM, Instituto de Higiene e Medicina Tropical, Universidade Nova de Lisboa, Rua da Junqueira 100, 1349-008 Lisboa, Portugal; 3 Centro de Recursos Microbiológicos (CREM), Faculdade de Ciências e Tecnologia, UNL, 2829-516 Caparica, Portugal; 4 Cost Action BM0701 (ATENS

## Abstract

**Background:**

Active efflux systems and reduced cell-wall permeability are considered to be the main causes of mycobacterial intrinsic resistance to many antimicrobials. In this study, we have compared the *Mycobacterium smegmatis *wild-type strain mc^2^155 with knockout mutants for porins MspA (the main porin of *M. smegmatis*) and MspC, the efflux pump LfrA (the main efflux pump system of *M. smegmatis*) and its repressor LfrR for their ability to transport ethidium bromide (EtBr) on a real-time basis. This information was then correlated with minimum inhibitory concentrations (MICs) of several antibiotics in the presence or absence of the efflux inhibitors chlorpromazine, thioridazine and verapamil.

**Results:**

In the absence of porins MspA and MspC, accumulation of ethidium bromide decreased and the cells became more resistant to several antibiotics, whereas the knockout mutant for the LfrA pump showed increased accumulation of EtBr and increased susceptibility to EtBr, rifampicin, ethambutol and ciprofloxacin. Moreover, the efflux inhibitors caused a reduction of the MICs of streptomycin, rifampicin, amikacin, ciprofloxacin, clarithromycin and erythromycin in most of the strains tested.

**Conclusions:**

The methodology used in this study demonstrated that porin MspA plays an important role in the influx of quaternary ammonium compounds and antibiotics and that efflux *via *the LfrA pump is involved in low-level resistance to several antimicrobial drugs in *M. smegmatis*. The results obtained with this non-pathogenic mycobacterium will be used in future studies as a model for the evaluation of the activity of the same efflux inhibitors on the susceptibility of multidrug resistant strains of *Mycobacterium tuberculosis *to isoniazid and rifampicin.

## Background

The intrinsic resistance of mycobacteria to most antimicrobial agents is generally attributed to their relatively impermeable cell-wall, which provides a barrier to noxious compounds and limits drug uptake [1]. This low permeability is due to the structure and lipid-rich composition of the mycobacterial cell-wall that comprises long-chain fatty acids, the mycolic acids, covalently bound to a peptidoglycan-arabinogalactan polymer, and extractable lipids not covalently linked to the peptidoglycan-arabinogalactan [1-3]. Diffusion of hydrophilic nutrients is mediated by pore-forming proteins like the MspA porin of *M. smegmatis*, which is described as the major diffusion pathway for hydrophilic solutes in these mycobacteria [4,5]. Along with the controlled permeability by the cell-wall, active efflux systems can also provide resistance by extruding noxious compounds prior to their reaching their intended targets. Intracellular concentration of a given compound is therefore a result of interplay between permeability and efflux [6]. In order to develop effective antimycobacterial therapeutic strategies at a time when multidrug resistant and extensively drug resistant tuberculosis continue to escalate [7], the contributions made by alterations of permeability due to down regulation of porins and increased expression of efflux pumps that render these infections problematic for therapy, must be understood.

Several mycobacterial efflux pumps have been identified and characterized to date [8-14]. However, their role in intrinsic and acquired drug resistance in mycobacteria is not completely understood. LfrA, a transporter protein of the major facilitator superfamily of *M. smegmatis*, was the first efflux pump to be genetically described in mycobacteria and it has been associated with resistance to ethidium bromide (EtBr), acriflavine, doxorubicin, rhodamine 123 and fluoroquinolones [14-17]. The regulation of LfrA is controlled by the upstream region of *lfrA *that contains a gene coding for LfrR, a putative transcriptional repressor of the TetR family, which represses the transcription of the *lfrRA *operon by directly binding to the promoter region [18,19].

The efflux pump substrate EtBr is widely used as a probe to detect and quantify efflux activity by bacteria [20-23]. EtBr emits weak fluorescence in aqueous solution (outside cells) and becomes strongly fluorescent when concentrated in the periplasm of Gram-negative bacteria and in the cytoplasm of Gram-positive bacteria. As long as EtBr is not intercalated between nucleic bases of DNA, it is subject to extrusion. When it is intercalated, the binding constant is sufficiently strong to keep EtBr from access to the efflux pump system of the bacterium [24]. Recently, a semi-automated fluorometric method was developed using EtBr as substrate for the real-time assessment of efflux pump activity in bacteria [25-27]. The method was developed considering that EtBr accumulation inside the cell is the result of the interplay between cell-wall permeability and efflux activity. The fluorescence that results from the overall intracellular EtBr content is monitored by real-time fluorometry.

In the study to be described, we used this semi-automated fluorometric method to study EtBr transport in *M. smegmatis*, using the wild-type strain mc^2^155 and mutant strains carrying in-frame deletions of genes coding for porins MspA and MspC, the efflux pump LfrA and its repressor LfrR, and correlated this information with the corresponding antibiotic profile. Since many efflux pumps of *M. smegmatis *have their homologues in *Mycobacterium tuberculosis*, the use of *M. smegmatis *as a model mycobacterium may provide data that will help to understand efflux-mediated drug resistance in *M. tuberculosis *and other mycobacteria that infect the human [15].

## Results and Discussion

### MspA as a major pathway for EtBr in *M. smegmatis*

**Figure 1:**
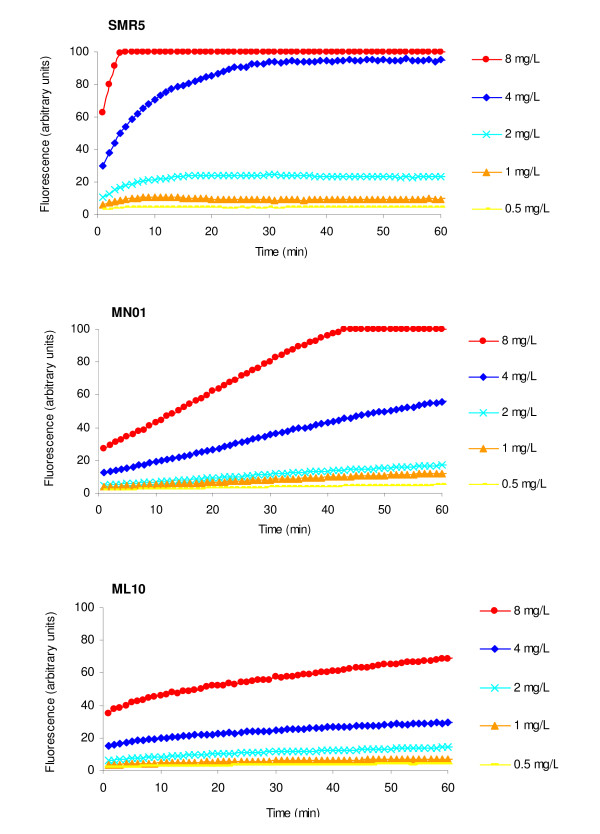
**Accumulation of increasing concentrations of EtBr (0.5-8 mg/L) by *M. smegmatis *SMR5, MN01 (Δ*mspA*) and ML10 (Δ*mspA*Δ*mspC*)**.

**Figure 2:**
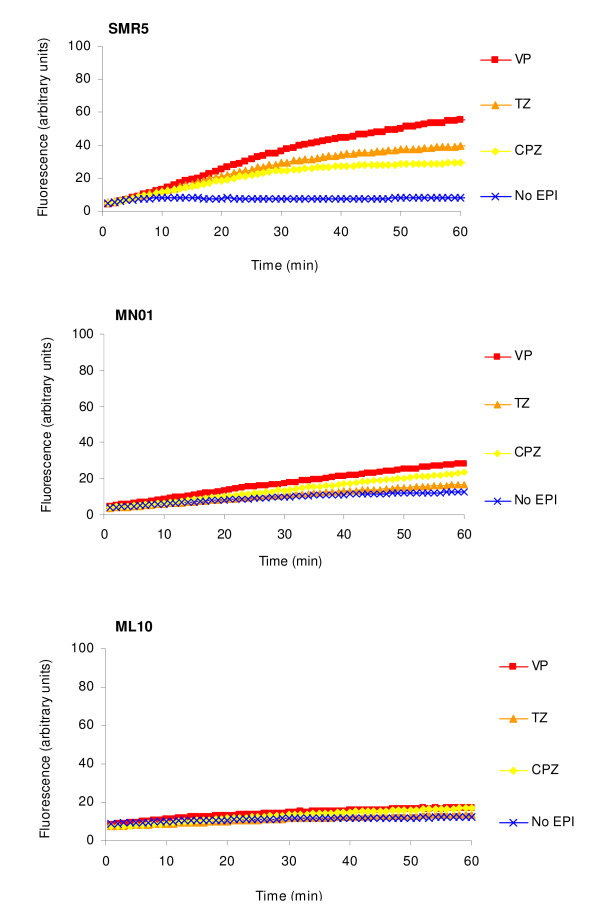
**Effect of efflux inhibitors on the accumulation of EtBr at 1, 2 and 4 mg/L by *M. smegmatis *SMR5, MN01 (Δ*mspA*) and ML10 (Δ*mspA*Δ*mspC*), respectively**. CPZ, chlorpromazine; EPI, efflux pump inhibitor; TZ, thioridazine; VP, verapamil.

The *M. smegmatis *strains used in this study are described in Table 1. The accumulation of increasing concentrations of EtBr by strains SMR5, MN01 (Δ*mspA*) and ML10 (Δ*mspA*ΔmspC) is presented by Figure 1. Accumulation of EtBr under conditions that maximize efflux (presence of glucose and incubation at 37°C) begins to take place at a concentration of 1 mg/L in the case of *M. smegmatis *SMR5. This concentration of EtBr marginally exceeds the ability of the intrinsic efflux system of SMR5 to extrude the substrate. In the case of the SMR5 derived porin mutants MN01 (Δ*mspA*) and ML10 (Δ*mspA *Δ*mspC*), the marginal concentration that results in accumulation of EtBr is increased to 2 and 4 mg/L, respectively (Figure 1) and considered to be the result of a decreased influx rate of EtBr due to the deletion of porins in these strains [3,5]. These concentrations were selected to test the effect of the efflux inhibitors chlorpromazine, thioridazine and verapamil in the accumulation of EtBr by these strains. This is to ensure that the increase of accumulation of EtBr is due to inhibition of efflux pumps and not to the use of an EtBr concentration that the cell's efflux system cannot extrude. As shown by Figure 2, the efflux inhibitors chlorpromazine, thioridazine and verapamil, used at ½ the minimum inhibitory concentration (MIC; see Table 1), increased accumulation of EtBr, although only marginally in strain ML10. We interpret these results as indicating that because of the absence of both porins in ML10, little EtBr enters the cell, accumulation does not take place, and hence, there is no EtBr subject for extrusion.

**Table 1:** Description of *M. smegmatis *strains used in this study and corresponding MICs determined for EtBr and efflux inhibitors

*M. smegmatis *strain	Description [Reference]	MICs (mg/L)			
		
		EtBr	CPZ	TZ	VP
**mc^2^155**	Wild-type [34]	6.25	25	12.5	200

**SMR5**	mc^2^155 derivative; resistant to streptomycin due to a mutation in ribosomal protein S12 (*rpsL*) [29]	6.25	25	12.5	400

**MN01**	SMR5 Δ*mspA *[5]	6.25	25	25	400

**ML10**	SMR5 Δ*mspA *Δ*mspC *[28]	12.5	25	25	250

**XZL1675**	mc^2^155 Δ*lfrA *[15]	0.4	25	6.25	125

**XZL1720**	mc^2^155 Δ*lfrR *[15]	6.25	25	12.5	200

CPZ, chlorpromazine; EtBr, ethidium bromide; TZ, thioridazine; VP, verapamil.

### LfrA is the main efflux system involved in EtBr extrusion in *M. smegmatis*

**Figure 3:**
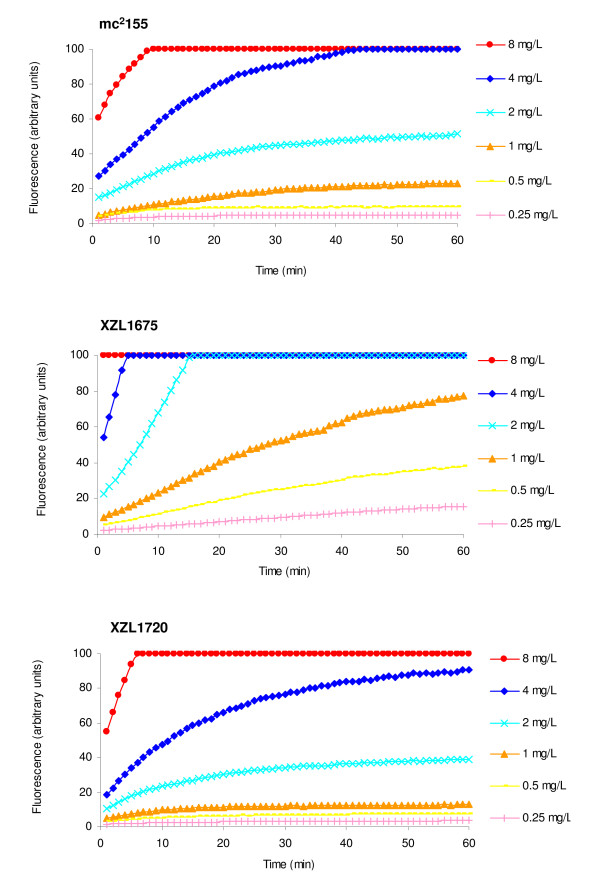
**Accumulation of increasing concentrations of EtBr (0.25-8 mg/L) by *M. smegmatis *mc^2^155, XZL1675 (Δ*lfrA*) and XZL1720 (Δ*lfrR*)**.

**Figure 4:**
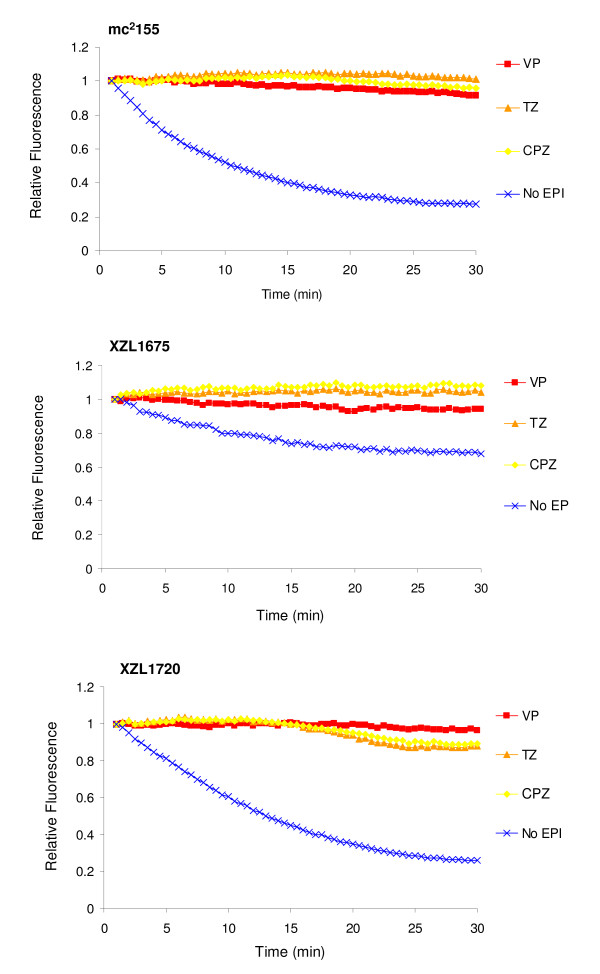
**Efflux of EtBr by *M. smegmatis *mc^2^155, XZL1675 (Δ*lfrA*) and XZL1720 (Δ*lfrR*)**. Efflux takes place at 37°C in the presence of glucose and is inhibited by the efflux inhibitors thioridazine and verapamil. EtBr was used at ½ MIC for each strain in order to ensure maximum EtBr-loading of the bacteria, without compromising cellular viability. CPZ, chlorpromazine; EPI, efflux pump inhibitor; TZ, thioridazine; VP, verapamil.

The accumulation of increasing concentrations of EtBr by strains mc^2^155, XZL1675 (Δ*lfrA*) and XZL1720 (Δ*lfrR*) is presented by Figure 3. Concerning the knockout mutant for the efflux pump LfrA (strain XZL1675), EtBr started to accumulate at a concentration of 0.25 mg/L. Since in the wild-type strain *M. smegmatis *mc^2^155, accumulation took place at a concentration of 1 mg/L of EtBr, these results demonstrate an increased susceptibility of the mutant strain to EtBr due to the inactivation of efflux pump LfrA. In the case of the *lfrR *knockout mutant XZL1720, EtBr accumulation started at a concentration of 2 mg/L, a higher concentration than the observed for the wild-type. This could be due to the constitutive expression of LfrA in this strain as a consequence of the deletion of its repressor, LfrR. These results are in agreement to what has been previously reported regarding LfrA as the main efflux system involved in EtBr extrusion [15-17]. In order to determine the effect of the efflux inhibitors chlorpromazine, thioridazine and verapamil on EtBr efflux activity, efflux assays were performed for *M. smegmatis *mc^2^155, XZL1675 and XZL1720. As shown by Figure 4, all strains presented efflux of EtBr at 37°C in the presence of glucose. Moreover, this efflux activity was inhibited by chlorpromazine, thioridazine and verapamil. However, the concentration of EtBr used for the *lfrA *mutant was 15-fold lower than the concentration used for the wild-type and *lfrR *deleted strains (0.2 mg/L for XZL1675 *vs *3 mg/L for mc^2^155 and XZL1720, ½ MIC for each strain - see Table 1). This further demonstrates that deletion of *lfrA *hinders the cell's ability to efflux EtBr, resulting in a low MIC for this fluorochrome and a decreased EtBr efflux activity when compared to mc^2^155 and XZL1720.

### Effect of efflux inhibitors on the antibiotic resistance of *M. smegmatis*

In order to correlate the data obtained from the fluorometric method with a drug susceptibility profile, the MICs of several antibiotics were determined for each strain (Table 2). Moreover, the effect of the efflux inhibitors on the reduction of MICs of the same antibiotics was also tested (Table 2). *M. smegmatis *SMR5, MN01 and ML10 present an MIC for streptomycin above 256 mg/L due to the presence of a mutation in the *rpsL *gene that confers resistance to this antibiotic [5,28,29]. Deletion of porins MspA (MN01) and MspC (ML10) caused a decreased susceptibility to clarithromycin, erythromycin and rifampicin. Deletion of *lfrA *(XZL1675) increased the susceptibility to ciprofloxacin and ethambutol (Table 2), which suggests that LfrA might contribute to the intrinsic resistance of *M. smegmatis *to these drugs, as already reported by other studies [15]. Moreover, the LfrA mutant also showed increased susceptibility to EtBr, thioridazine and verapamil (Table 1).

**Table 2:** Effect of efflux inhibitors on the MICs of antibiotics for wild-type and mutant strains of *M. smegmatis*

MICs (mg/L)							
		***M. smegmatis *strains**					
		
**Antibiotic/EPI**		**mc^2^155 (wild-type)**	**SMR5 (mc^2^155 STR^r^)**	**MN01 (SMR5 Δ*mspA*)**	**ML10 (SMR5 Δ*mspA *Δ*mspC*)**	**XZL1675 (mc^2^155 Δ*lfrA*)**	**XZL1720 (mc^2^155 Δ*lfrR*)**

	No EPI	0.5	0.5	0.5	0.5	0.5	0.5
	
**AMK**	CPZ	**0.125**	**0.125**	**0.125**	0.25	**0.063**	**0.063**
	
	TZ	**0.063**	**0.063**	**0.125**	0.25	**0.063**	**0.063**
	
	VP	**0.125**	**0.125**	**0.125**	0.25	**0.125**	**0.125**

	No EPI	0.25	0.25	0.25	0.25	0.125	0.125
	
**CIP**	CPZ	**0.063**	**0.063**	**0.063**	**0.063**	0.063	0.063
	
	TZ	**0.063**	**0.063**	**0.063**	**0.063**	**0.032**	**0.032**
	
	VP	**0.063**	**0.063**	**0.063**	**0.063**	0.063	0.063

	No EPI	2	2	8	8	2	2
	
**CLT**	CPZ	**0.25**	**0.25**	**0.5**	**1**	**0.25**	**0.25**
	
	TZ	**0.25**	**0.25**	**1**	**1**	**0.25**	**0.25**
	
	VP	**0.5**	**0.5**	**0.5**	**1**	**0.5**	**0.5**

	No EPI	1	1	1	1	0.5	1
	
**EMB**	CPZ	1	1	1	1	0.5	1
	
	TZ	1	1	1	1	0.5	1
	
	VP	1	1	1	1	0.5	1

	No EPI	32	32	64	64	32	32
	
**ERY**	CPZ	**4**	**4**	**8**	**8**	**4**	**4**
	
	TZ	**4**	**4**	**16**	**16**	**4**	**4**
	
	VP	**8**	**8**	**8**	**8**	**8**	**8**

	No EPI	4	4	8	8	0.5	0.5
	
**RIF**	CPZ	**1**	**1**	**2**	**2**	**0.125**	**0.125**
	
	TZ	2	2	4	4	**0.125**	**0.125**
	
	VP	2	2	4	4	**0.125**	0.25

	No EPI	0.5	>256	>256	>256	0.5	0.5
	
**STR**	CPZ	**0.125**	>256	>256	>256	**0.032**	**0.063**
	
	TZ	**0.125**	>256	>256	>256	**0.125**	0.25
	
	VP	0.25	>256	>256	>256	0.25	**0.125**

AMK, amikacin; CIP, ciprofloxacin; CLT, clarithromycin; CPZ, chlorpromazine; EMB, ethambutol; EPI, efflux pump inhibitor; ERY, erythromycin; RIF, rifampicin; STR, streptomycin; TZ, thioridazine; VP, verapamil. Data in bold type represents significant (at least 4-fold) reduction of the MIC produced by the presence of an efflux inhibitor.

Relatively to the effect of the efflux inhibitors on the MICs of the tested antibiotics, there is an overall reduction of the MICs, with the exception of ethambutol, in all of the studied strains. The fact that the effect of these inhibitors is not dependent of a given genotype suggests that these compounds have a wide range of activity against efflux and are not specific of a particular efflux pump.

Some of the results obtained in this study are at variance with those reported by others. Firstly, the previous characterization of the *lfrA *and *lfrR *knockout mutant strains by Li and Nikaido [15] showed that there is no difference between the mutant strains and the wild-type concerning the MIC for rifampicin (authors reported an MIC of 1 mg/L for each strain). In our study, we observed a decrease of the MIC against the *lfrA *and *lfrR *deleted mutants. Secondly, whereas deletion of *lfrR *is reported to increase the ciprofloxacin MIC from 0.25 mg/L (wild-type) to 4 mg/L (XZL1720) [15], our results show that the MIC for ciprofloxacin against the *lfrR *mutant is the same observed for the *lfrA *mutant. The variance between our results and those of others may be due to the use of different methods for the determination of the MICs: microdilution method in Middlebrook 7H9 medium supplemented with oleic acid albumin dextrose catalase (OADC) (this study) or microdilution method in Middlebrook 7H9 medium supplemented with OADC and Tween 80 in combination with drug gradient plates [15].

## Conclusions

The detection of EtBr influx and efflux can be used to anticipate transport-mediated antibiotic resistance in bacteria, since some of these compounds use similar channels to enter and leave the cell. In this study, we have compared the wild-type *M. smegmatis *mc^2^155 with knockout mutants for LfrA and MspA for their ability to transport EtBr. It was observed that in the absence of MspA, the major porin of *M. smegmatis*, accumulation of EtBr decreased and the mycobacteria became more resistant to several antibiotics. This is in accordance with previous studies that demonstrated MspA as the major diffusion pathway for hydrophilic solutes in *M. smegmatis*, mediating the uptake of small and hydrophilic nutrients such as sugars and phosphates across the outer membrane [4,28,30]. Permeability of the cell to EtBr is, in our opinion, dependent for the most part on the presence of the major porin MspA. If this were not so, we would then expect little difference in the accumulation between intact and MspA deficient strains. This conclusion is supported by others that demonstrated that deletion of the *mspA *gene increased the resistance of *M. smegmatis *not only to hydrophilic molecules, but also to hydrophobic antibiotics, such as erythromycin [31]. However, deletion of *mspA *causes the alteration in the organisation of lipids of the mycobacterial outer membrane, resulting in a decreased rate of uptake of hydrophobic agents such as chenodeoxycholate [31,32]. In fact, it has been previously demonstrated that a *M. tuberculosis *mutant lacking oxygenated mycolic acids also presents altered lipid organisation within its outer membrane, and the permeability to various agents is also altered [31,32]. Undoubtedly, the lipid organisation and lipid composition of the outer membrane of mycobacteria significantly affects the permeability of agents into the cell.

The mutant for the LfrA pump showed increased accumulation of EtBr and increased susceptibility to EtBr, ethambutol and ciprofloxacin. This is in agreement with other studies that showed that disruption of the *lfrA *gene decreased the MIC of EtBr, acriflavine, ciprofloxacin, doxorubicin and rhodamine [13,16]. Moreover, it was shown that resistance to the tested antibiotics decreased in the presence of efflux inhibitors in the studied strains, demonstrating that these inhibitors have a broad range of activity that is not specific to a given genotype.

In conclusion, the methodology used in this study demonstrates that porin MspA plays an important role in the entrance of quaternary ammonium compounds and antibiotics into the cell. Whether its absence is the main cause for decreased permeability, or that its absence has resulted in altered lipid structure of the outer membrane that is less permeable remains to be elucidated. The same methodology used to assess permeability also assessed the activity of the main efflux pump LfrA of the wild-type strain and of LfrA and LfrR depleted mutants and correlated the degree of activity with low-level resistance to several antimicrobial drugs.

The methodology used and the results obtained in this work will be used in future studies as a working model for the evaluation of influx and efflux of substrates by multidrug resistant *M. tuberculosis *clinical isolates and, therefore, determine the cause for the multidrug resistant phenotype beyond simple mutation of relevant targets.

## Methods

### Materials

EtBr, glucose, phosphate buffered solution (PBS), chlorpromazine, thioridazine, verapamil, amikacin, ciprofloxacin, ethambutol, erythromycin, rifampicin and streptomycin were purchased from Sigma Aldrich Química SA (Madrid, Spain). Clarithromycin was obtained from Abbott Laboratories (Abbott Park, IL, USA). Middlebrook 7H9 broth and OADC supplement were purchased from Difco (Detroit, MI, USA). All solutions were prepared on the day of the experiment.

### Bacteria

The *M. smegmatis *strains used in this work are described in Table 1. *M. smegmatis *strains SMR5, MN01 and ML10 were kindly provided by Michael Niederweis (Department of Microbiology, University of Alabama at Birmingham, Birmingham, U.S.A); strains XZL1675 and XZL1720 were kindly provided by Hiroshi Nikaido (Department of Molecular and Cell Biology, University of California, Berkeley, California, U.S.A). Mycobacteria were grown at 37°C in Middlebrook 7H9 broth or Middlebrook 7H11 solid medium, supplemented with 10% (v/v) of OADC.

### Determination of Minimum Inhibitory Concentrations

The determination of MICs of EtBr, the efflux inhibitors chlorpromazine, thioridazine and verapamil and of antibiotics studied alone and in the presence of an efflux inhibitor, was performed by the broth microdilution method according to the CLSI guidelines [33]. Briefly, mycobacterial strains were grown at 37°C in Middlebrook 7H9 broth supplemented with 10% OADC until an optical density (O.D.) of 0.8 at a wavelength of 600 nm. The number of colony-forming units (cfu) corresponding to aliquots of the inoculum was routinely calculated in order to ensure a constant number of bacterial cells from experiment to experiment. Bacterial cultures were diluted in PBS to equal the McFarland No. 0.5 standard and the final inoculum was prepared by diluting the bacterial suspension at 1:100. Aliquots of 0.1 mL were transferred to each well of a 96-well plate that contained 0.1 mL of each compound at concentrations prepared from 2-fold serial dilutions in 7H9/OADC medium. The inoculated plates were incubated at 37°C until growth in the agent-free control-well was evident (2-3 days). The MIC was defined as the lowest concentration of compound that inhibited visible growth.

### Semi-automated fluorometric method

The assessment of accumulation and extrusion of EtBr on a real-time basis by *M. smegmatis *strains wild-type mc^2^155, SMR5, porin mutants, MN01 and ML10 and efflux mutants XZL1675 and XZL1720 (Table 1) was performed using the semi-automated fluorometric method, as previously described [25-27].

#### (i) Accumulation assay

*M. smegmatis *strains were grown in 5 mL of 7H9/OADC medium at 37°C until an O.D._600 _of 0.8. Cultures were centrifuged at 13000 rpm for 3 minutes, the supernatant discarded and the pellet washed in PBS (pH 7.4). The O.D._600 _was adjusted to 0.4 with PBS and glucose was added at final concentration of 0.4%. Aliquots of 0.095 mL of bacterial suspension were distributed to 0.2 mL PCR microtubes and EtBr was added at concentrations that ranged from 0.25 to 8 mg/L. Fluorescence was measured in the Rotor-Gene™ 3000 (Corbett Research, Sydney, Australia), using the 530 nm band-pass and the 585 nm high-pass filters as the excitation and detection wavelengths, respectively. Fluorescence data was acquired every 60 seconds for 60 minutes at 37°C.

The effect of chlorpromazine, thioridazine and verapamil on the accumulation of EtBr was determined by adding 0.005 mL of each compound to aliquots of 0.095 mL of EtBr-containing bacterial suspension previously distributed to 0.2 mL PCR microtubes. Fluorescence was measured every 60 seconds for 60 minutes at 37°C in the Rotor-Gene™ 3000. Each inhibitor was used at ½ the MIC in order to not compromise the cellular viability (as confirmed by CFUs counting).

#### (ii) Efflux assay

Mycobacteria were exposed to conditions that promote maximum accumulation of EtBr: EtBr at ½ MIC for each strain; no glucose; presence of the efflux inhibitor that caused maximum accumulation, in this case verapamil; and incubation at 25°C [25-27]. The EtBr loaded cells were centrifuged at 13000 rpm for 3 minutes and resuspended in EtBr-free PBS containing 0.4% glucose. After adjusting the O.D._600 _to 0.4, aliquots of 0.095 mL were transferred to 0.2 mL microtubes. Fluorescence was measured in the Rotor-Gene™ 3000 as described for the accumulation assay. Efflux activity was quantified by comparing the fluorescence data obtained under conditions that promote efflux (presence of glucose and absence of efflux inhibitor) with the data from the control in which the mycobacteria are under conditions of no efflux (presence of an inhibitor and no energy source). Thus, the relative fluorescence corresponds to the ratio of fluorescence that remains per unit of time, relatively to the EtBr-loaded cells.

## Authors' contributions

LR designed the experiments, carried out the EtBr accumulation and efflux assays and drafted the manuscript. JR performed the MIC determination assays and participated in the EtBr efflux assays. IC participated in the study design and coordination and helped to draft the manuscript. LA participated in the study design and revised the manuscript. MV conceived of the study, participated in its design and coordination and helped to draft the manuscript. All authors read and approved the final manuscript.

## References

[B1] Brennan PJ Nikaido H The envelope of mycobacteria Annu Rev Biochem 1995 64 29 63 10.1146/annurev.bi.64.070195.000333 7574484

[B2] Brennan PJ Structure, function, and biogenesis of the cell wall of *Mycobacterium tuberculosis* Tuberculosis 2003 83 91 97 10.1016/S1472-9792(02)00089-6 12758196

[B3] Niederweis M Mycobacterial porins - new channel proteins in unique outer membranes Mol Microbiol 2003 49 1167 1177 10.1046/j.1365-2958.2003.03662.x 12940978

[B4] Niederweis M Ehrt S Heinz C Klöcker U Karosi S Swiderek KM Riley LW Benz R Cloning of the *mspA *gene encoding a porin from *Mycobacterium smegmatis* Mol Microbiol 1999 33 933 945 10.1046/j.1365-2958.1999.01472.x 10476028

[B5] Stahl C Kubetzko S Kaps I Seeber S Engelhardt H Niederweis M MspA provides the main hydrophilic pathway through the cell wall of *Mycobacterium smegmatis* Mol Microbiol 2001 40 451 464 10.1046/j.1365-2958.2001.02394.x 11309127

[B6] Nikaido H Preventing drug access to targets: cell surface permeability barriers and active efflux in bacteria Semin Cell Dev Biol 2001 12 215 23 10.1006/scdb.2000.0247 11428914

[B7] World Health Organization Multidrug and extensively drug-resistant TB (M/XDR-TB): 2010 global report on surveillance and response 2010 Geneva, Switzerland

[B8] Aínsa JA Blokpoel MC Otal I Young DB De Smet KA Martín C Molecular cloning and characterization of Tap, a putative multidrug efflux pump present in *Mycobacterium fortuitum *and *Mycobacterium tuberculosis* J Bacteriol 1998 180 5836 5843 9811639 10.1128/jb.180.22.5836-5843.1998 PMC107655

[B9] Choudhuri BS Bhakta S Barik R Basu J Kundu M Chakrabarti P Overexpression and functional characterization of an ABC (ATP-binding cassette) transporter encoded by the genes *drrA *and *drrB *of *Mycobacterium tuberculosis* Biochem J 2002 367 279 285 10.1042/BJ20020615 12057006 PMC1222852

[B10] De Rossi E Aínsa JA Riccardi G Role of mycobacterial efflux transporters in drug resistance: an unresolved question FEMS Microbiol Rev 2006 30 36 52 10.1111/j.1574-6976.2005.00002.x 16438679

[B11] Siddiqi N Das R Pathak N Banerjee S Ahmed N Katoch VM Hasnain SE *Mycobacterium tuberculosis *isolate with a distinct genomic identity overexpresses a *tap*-like efflux pump Infection 2004 32 109 111 10.1007/s15010-004-3097-x 15057575

[B12] Ramón-García S Martín C Thompson CJ Aínsa JA Role of the *Mycobacterium tuberculosis *P55 efflux pump in intrinsic drug resistance, oxidative stress responses, and growth Antimicrob Agents Chemother 2009 53 3675 3682 19564371 10.1128/AAC.00550-09 PMC2737831

[B13] Takiff HE Cimino M Musso MC Weisbrod T Martinez R Delgado MB Salazar L Bloom BR Jacobs WR Jr Efflux pump of the proton antiporter family confers low-level fluoroquinolone resistance in *Mycobacterium smegmatis* Proc Natl Acad Sci USA 1996 93 362 366 10.1073/pnas.93.1.362 8552639 PMC40238

[B14] Viveiros M Leandro C Amaral L Mycobacterial efflux pumps and chemotherapeutic implications Int J Antimicrob Agents 2003 22 274 278 10.1016/S0924-8579(03)00208-5 13678834

[B15] Li XZ Zhang L Nikaido H Efflux pump-mediated intrinsic drug resistance in *Mycobacterium smegmatis* Antimicrob Agents Chemother 2004 48 2415 2423 10.1128/AAC.48.7.2415-2423.2004 15215089 PMC434187

[B16] Liu J Takiff HE Nikaido H Active efflux of fluoroquinolones in *Mycobacterium smegmatis *mediated by LfrA, a multidrug efflux pump J Bacteriol 1996 178 3791 3795 8682782 10.1128/jb.178.13.3791-3795.1996 PMC232638

[B17] Sander P De Rossi E Böddinghaus B Cantoni R Branzoni M Böttger EC Takiff H Rodriquez R Lopez G Riccardi G Contribution of the multidrug efflux pump LfrA to innate mycobacterial drug resistance FEMS Microbiol Lett 2000 193 19 23 10.1111/j.1574-6968.2000.tb09396.x 11094273

[B18] Bellinzoni M Buroni S Schaeffer F Riccardi G De Rossi E Alzari PM Structural plasticity and distinct drug-binding modes of LfrR, a mycobacterial efflux pump regulator J Bacteriol 2009 191 7531 7537 10.1128/JB.00631-09 19820093 PMC2786614

[B19] Buroni S Manina G Guglierame P Pasca MR Riccardi G De Rossi E LfrR is a repressor that regulates expression of the efflux pump LfrA in *Mycobacterium smegmatis* Antimicrob Agents Chemother 2006 50 4044 4052 10.1128/AAC.00656-06 17043130 PMC1694004

[B20] Jernaes MW Steen HB Staining of *Escherichia coli *for flow cytometry: influx and efflux of ethidium bromide Cytometry 1994 17 302 309 10.1002/cyto.990170405 7875037

[B21] Greulich KO Single molecule techniques for biomedicine and pharmacology Curr Pharm Biotechnol 2004 5 243 259 10.2174/1389201043376878 15180546

[B22] Martins M Santos B Martins A Viveiros M Couto I Cruz A Pagès JM Molnar J Fanning S Amaral L Management Committee Members of Cost B16 European Commission/European Science Foundation An instrument-free method for the demonstration of efflux pump activity of bacteria In Vivo 2006 20 657 664 17091774

[B23] Schumacher A Trittler R Bohnert JA Kümmerer K Pagès JM Kern WV Intracellular accumulation of linezolid in *Escherichia coli*, *Citrobacter freundii *and *Enterobacter aerogenes*: role of enhanced efflux pump activity and inactivation J Antimicrob Chemother 2007 59 1261 1264 10.1093/jac/dkl380 16971414

[B24] Sharples D Brown JR Correlation of the base specificity of DNA-intercalating ligands with their physico-chemical properties FEBS Lett 1976 69 37 40 10.1016/0014-5793(76)80648-5 1033085

[B25] Rodrigues L Wagner D Viveiros M Sampaio D Couto I Vavra M Kern WV Amaral L Thioridazine and chlorpromazine inhibition of ethidium bromide efflux in *Mycobacterium avium *and *Mycobacterium smegmatis* J Antimicrob Chemother 2008 61 1076 1082 10.1093/jac/dkn070 18310137

[B26] Viveiros M Martins A Paixão L Rodrigues L Martins M Couto I Fähnrich E Kern WV Amaral L Demonstration of intrinsic efflux activity of *Escherichia coli *K-12 AG100 by an automated ethidium bromide method Int J Antimicrob Agents 2008 31 458 462 10.1016/j.ijantimicag.2007.12.015 18343640

[B27] Viveiros M Rodrigues L Martins M Couto I Spengler G Martins A Amaral L S. H. Gillespie Evaluation of efflux activity of bacteria by a semi-automated fluorometric system Antibiotic Resistance Methods and Protocols (Methods in Molecular Medicine) 2010 642 2 New York: Humana Press 159 172 10.1007/978-1-60327-279-7_12 20401593

[B28] Stephan J Bender J Wolschendorf F Hoffmann C Roth E Mailänder C Engelhardt H Niederweis M The growth rate of *Mycobacterium smegmatis *depends on sufficient porin-mediated influx of nutrients Mol Microbiol 2005 58 714 730 10.1111/j.1365-2958.2005.04878.x 16238622

[B29] Sander P Meier A Böttger EC *rpsL*+: a dominant selectable marker for gene replacement in mycobacteria Mol Microbiol 1995 16 991 1000 10.1111/j.1365-2958.1995.tb02324.x 7476195

[B30] Wolschendorf F Mahfoud M Niederweis M Porins are required for uptake of phosphates by *Mycobacterium smegmatis* J Bacteriol 2007 189 2435 2442 10.1128/JB.01600-06 17209034 PMC1899398

[B31] Stephan J Mailaender C Etienne G Daffé M Niederweis M Multidrug resistance of a porin deletion mutant of *Mycobacterium smegmatis* Antimicrob Agents Chemother 2004 48 4163 4170 10.1128/AAC.48.11.4163-4170.2004 15504836 PMC525411

[B32] Dubnau E Chan J Raynaud C Mohan VP Laneelle MA Yu K Quemard A Smith I Daffé M Oxygenated mycolic acids are necessary for virulence of *Mycobacterium tuberculosis *in mice Mol. Microbiol 2000 36 630 637 10.1046/j.1365-2958.2000.01882.x 10844652

[B33] Clinical and Laboratory Standards Institute (CLSI) Susceptibility Testing of Mycobacteria, Nocardiae, and Other Aerobic Actinomycetes; Approved Standard. CLSI M24-A 2003 Wayne, PA 31339680

[B34] Snapper SB Melton RE Mustafa S Kieser T Jacobs WR Jr Isolation and characterization of efficient plasmid transformation mutants of *Mycobacterium smegmatis* Mol Microbiol 1990 4 1911 1919 10.1111/j.1365-2958.1990.tb02040.x 2082148

